# Impact of Meditation Device Use on Quality of Life of Patients Following Surgery for Hypercortisolism

**DOI:** 10.1210/jendso/bvaf145

**Published:** 2025-09-03

**Authors:** Jasmine Saini, Elio Ferreira Taveras, Yana Hleibiehova, Bahar Bahrani Fard, Malavika Suresh, Rashi Sandooja, Bahaa H Salama, Vanessa Fell, Elizabeth J Atkinson, Sara J Achenbach, Irina Bancos

**Affiliations:** Division of Endocrinology and Metabolism, Department of Medicine, Mayo Clinic, Rochester, MN 55905, USA; Department of Internal Medicine, Yale New Haven Hospital, Yale School of Medicine, New Haven, CT 06520, USA; Division of Endocrinology and Metabolism, Department of Medicine, Mayo Clinic, Rochester, MN 55905, USA; Division of Endocrinology and Metabolism, Department of Medicine, Mayo Clinic, Rochester, MN 55905, USA; Division of Endocrinology and Metabolism, Department of Medicine, Mayo Clinic, Rochester, MN 55905, USA; Division of Endocrinology and Metabolism, Department of Medicine, Mayo Clinic, Rochester, MN 55905, USA; Department of Internal Medicine, Banner Wyoming Medical Center, Casper, WY 82601, USA; Division of Endocrinology and Metabolism, Department of Medicine, Mayo Clinic, Rochester, MN 55905, USA; Division of Endocrinology and Metabolism, Department of Medicine, University of Nebraska Medical Center, Omaha, NE 68198, USA; Division of Endocrinology and Metabolism, Department of Medicine, Mayo Clinic, Rochester, MN 55905, USA; Division of Endocrinology and Metabolism, Department of Medicine, Mayo Clinic, Rochester, MN 55905, USA; Division of Clinical Trials and Biostatistics, Department of Quantitative Health Sciences, Mayo Clinic, Rochester, MN 55905, USA; Division of Clinical Trials and Biostatistics, Department of Quantitative Health Sciences, Mayo Clinic, Rochester, MN 55905, USA; Division of Endocrinology and Metabolism, Department of Medicine, Mayo Clinic, Rochester, MN 55905, USA; Department of Laboratory Medicine and Pathology, Mayo Clinic, Rochester, MN 55905, USA

**Keywords:** MUSE, MUSE-2, Cushing, MACS, headband, adrenal, pituitary, recovery

## Abstract

**Context:**

Patients with endogenous hypercortisolism experience glucocorticoid withdrawal syndrome (GWS) after surgery. Meditation may be an effective intervention to alleviate the severity of GWS.

**Objective:**

To determine the acceptability of a portable, wearable electroencephalography device for guided meditation (MUSE headband) and the impact of MUSE use on GWS and quality of life 12 weeks postsurgery.

**Methods:**

We conducted a single-center prospective cohort study of adults with endogenous hypercortisolism undergoing curative surgery from 2019 to 2024. Patients had baseline and postsurgical assessments over 12 weeks. The study comprised patients using MUSE for ≥ 6 weeks (MUSE cohort) and patients matched by age, sex, BMI, hypercortisolism type, and glucocorticoid type at 1:4 ratio. Quality of life and GWS symptoms were assessed with AddiQoL, CushingQoL, and 36-item Short Form Health Survey mental and physical component (SF-36 MCS and PCS) questionnaires.

**Results:**

MUSE was offered to 52 patients, and 22 (42%) used MUSE for ≥ 6 weeks within 12 weeks after surgery. At baseline, compared to 88 matched subjects, 22 MUSE participants demonstrated similar prevalence of comorbidities and clinical and biochemical hypercortisolism severity, but lower AddiQoL (mean 73 vs 66, *P* = .031) and SF-36 MCS (mean 39 vs 33, *P* = .022). At 12 weeks, these differences in quality of life were no longer present. After adjusting for age, sex, BMI, clinical severity score, and baseline quality of life, MUSE use was an independent predictor of improved SF-36 PCS at 12 weeks postsurgery (beta 4.2, 95% CI: 0.5-7.9, *P* = .026).

**Conclusion:**

Postsurgical meditation intervention may improve physical symptoms and accelerate recovery.

Glucocorticoid withdrawal syndrome (GWS) is characterized by a constellation of symptoms that follow a sudden withdrawal of supraphysiologic glucocorticoid exposure, such as after successful surgery for endogenous hypercortisolism or during a rapid exogenous glucocorticoid taper [1-4]. Most commonly, patients report musculoskeletal pain, fatigue, mood changes, headache, poor concentration, sweating, and nausea [1, 5, 6]. We have previously shown that some symptoms of GWS worsen as glucocorticoid taper progresses toward 5 to 8 weeks after surgery [1].

Currently, there are no known evidence-based interventions to improve GWS. In the recent guidelines on glucocorticoid-induced adrenal insufficiency, authors suggested counseling, education on the adrenal insufficiency management, and a slower glucocorticoid taper [3]. Others suggested considering supportive therapy with antidepressants, pain management, and physical and behavioral therapy [2, 5, 7].

Meditation is a potential intervention to treat GWS. Meditation was reported to decrease stress and improve concentration, quality of life, depression, and sleep quality in patients with various nonendocrine disorders [8-11]. In patients with breast cancer, meditation positively impacted postsurgical recovery, with improvements in fatigue, quality of life, stress, and pain—symptoms similar to GWS [12, 13]. No studies to date investigated meditation in patients recovering from Cushing syndrome or mild autonomous cortisol secretion (MACS).

We took advantage of an ongoing prospective longitudinal study of patients with Cushing syndrome and MACS undergoing surgery to investigate the impact of the intervention with meditation on postsurgical recovery. We aimed (i) to determine the acceptability and use of a portable, wearable electroencephalography (EEG) device for guided meditation (MUSE headband); and (ii) to determine the impact of MUSE use on the trajectory of GWS and quality of life within 3 months postsurgery.

## Methods

### Study Design

We conducted a single-center prospective cohort study between August 6, 2019, and September 9, 2024. This study was approved by the institutional Review Board, IRB number #19006003. Study procedures were performed after explaining and obtaining informed consent from all participants and carried out in compliance with the Declaration of Helsinki.

### Participant Selection

**Figure 1.**
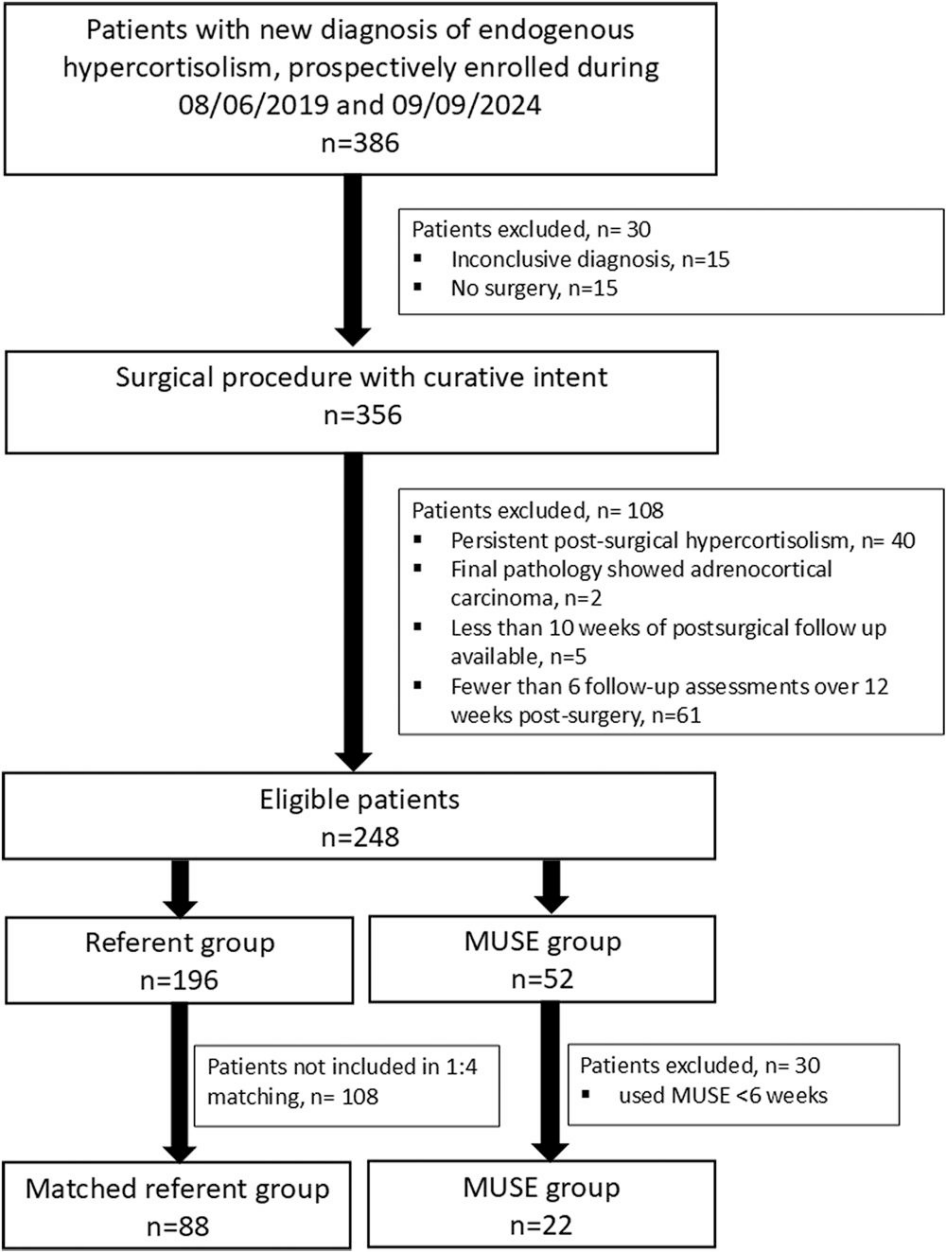
Flowchart showing participant inclusion.

Adults with endogenous hypercortisolism (adrenal, pituitary, or ectopic) were enrolled in this study. Only patients with clear diagnosis and treated with successful surgery were included in this substudy. Those with persistent postsurgical hypercortisolism, adrenal malignancy, and insufficient follow-up were excluded, Fig. 1. MUSE was offered to all eligible consecutive patients in 2 periods: (i) between May 1, 2020 and November 30, 2020; and (ii) between August 1, 2022 and April 30, 2024. Patients who used MUSE < 6 weeks during the 12 weeks after surgery were excluded from the final analysis. The referent group was selected from the 196 eligible patients with endogenous hypercortisolism at 1:4 ratio, matched by age, sex, body mass index (BMI), hypercortisolism type, clinical severity score, and glucocorticoid type (prednisone or hydrocortisone). Glucocorticoid taper with hydrocortisone and prednisone was standardized (see Supplemental data [14]).

### Meditation Intervention With MUSE-2 Headband

The MUSE-2 headband (InteraXon Inc, Toronto, Ontario) is a portable, wearable, wireless, headband-style EEG device for guided meditation (https://choosemuse.com). The device is designed to interact with a smartphone and is used in combination with the MUSE app. MUSE device converts the EEG signals measured over frontal and temporal cerebral cortices into measures of brain state and provides immediate real-time performance feedback through weather-related cues (gentle audio sounds). MUSE-2 headband system offers meditation sessions that can selectively vary in length, and has been previously reported to improve mood, anxiety, focus, and productivity [15]. Participants can choose various meditation type sessions, including Body, Breath, Heart, and Mind meditation [15, 16]. All patients were provided with a MUSE headband and instructions for use. The MUSE app was downloaded on their phone/tablet. MUSE headband was positioned on the patient's head to ensure fit, and a demonstration for 5-minute meditation was performed. Patients were instructed to use the MUSE device for at least 10 minutes each day for 3 months after the surgery. The meditation data collected was uploaded to a HIPPA-approved cloud server. Compliance was confirmed through weekly surveys and cloud server data. MUSE session of at least 55 seconds in duration was counted sufficient.

### Assessments

Patients in both groups (MUSE and referent group) were interviewed at baseline, and symptoms, physical examination, comorbidities, and hormonal workup were recorded in Redcap. Cushing syndrome disease severity was classified based on clinical and biochemical severity scores [17] (Supplemental data [14]). All participants underwent assessments for GWS and quality of life before and at 12 weeks postsurgery.

### Glucocorticoid Withdrawal Syndrome

Symptoms of GWS were assessed with the AddiQoL survey, as currently, there are no validated assessments for GWS, Supplemental data [14]. For symptoms with overlapping components such as fatigue, myalgia, arthralgia, inability to concentrate, and mood changes, a positive diagnosis required that these symptoms be present at least 50% of the time. We considered that a higher AddiQoL score reflected fewer symptoms of GWS and a lower AddiQoL score reflected more symptoms of GWS. A positive change in AddiQoL indicated improvement in GWS.

### Quality of Life

Quality of life was assessed using the 36-item Short Form Health Survey (SF-36) and CushingQoL surveys [18, 19]. SF-36 includes physical (physical functioning, role-physical limitation, bodily pain, general health) and mental (vitality, social functioning, role-emotional limitation, and mental health) domains that are combined to generate physical and mental component summary scores (Supplemental data [14]). Physical, psychosocial, and overall Cushing scores were calculated using the CushingQoL questionnaire, Supplemental data [14]. Higher scores on SF-36 and CushingQoL surveys indicated better quality of life.

### Outcomes

Outcomes included SF-36 (physical and mental component summary), AddiQoL score, CushingQoL.

### Statistics

Continuous variables were expressed as median and interquartile range (Q1-Q3) or mean (SD) and groups were compared using the Wilcoxon rank sum test or 2-sample *t* test as appropriate. Analysis of covariance models were used to evaluate group differences at 12 weeks after adjusting for baseline values. Categorical variables were expressed as counts and proportions and group differences were assessed using the chi-square test. Multivariable linear regression models were used to predict factors for successful response to MUSE at 12 weeks after surgery using a predefined variable set including adjustment for baseline values. A 2-tailed *P* value of <.05 was considered statistically significant for all tests. Statistical analysis was conducted using R version 4.4.1.

## Results

### Baseline

Of 386 patients prospectively enrolled in the study, 248 were eligible for inclusion (Fig. 1). Of 248 patients, MUSE was offered to 52 study participants, and 22 (42%) used MUSE for ≥ 6 weeks within 12 weeks after surgery. Of the referent cohort of 196 eligible patients, 88 patients were matched to the 22 MUSE participants (Fig. 1).

Patients in both referent and MUSE cohorts demonstrated similar demographics, BMI, type of hypercortisolism, prevalence and severity of comorbidities, clinical and biochemical hypercortisolism severity scores, hormonal measurements, and postsurgery glucocorticoid management (Table 1). However, patients in the MUSE cohort had lower quality of life at baseline (before surgery) in the mental component summary score of SF-36 and AddiQoL total score (Table 2).

**Table 1.** Baseline presurgical characteristics of participants

Characteristic	Referent cohort	MUSE cohort	*P* value
n	88	22	
**Demographic characteristics**			
Age at baseline, years	53.4	53.2	.642
Median (Q1-Q3)	(42.1-61.1)	(44.5-63.2)	
Women, n (%)	67 (76.1)	17 (77.3)	.911
White race/ethnicity, n (%)	77 (87.5)	20 (90.9)	.658
**Clinical assessment and physical examination findings**			
BMI, kg/m^2^	30.7	31.4	.881
Median (Q1-Q3)	(26.4-36.7)	(26.1-40.1)	
Type of hypercortisolism, n (%)			.993
MACS	34 (38.6)	8 (36.4)	
Adrenal Cushing	17 (19.3)	4 (18.2)	
Pituitary Cushing	33 (37.5)	9 (40.9)	
Ectopic Cushing	4 (4.5)	1 (4.5)	
Duration of symptoms before diagnosis, months	12.0	15.0	.231
Median (Q1-Q3)	(2.8-39.0)	(4.2-69.0)	
Hypertension, n (%)	71 (80.7)	19 (86.4)	.537
Hypertension treated with 3 or more antihypertensives, n (%)	31 (35.2)	7 (31.8)	.765
Hyperglycemia, n (%)			.822
Prediabetes	25 (28.4)	6 (27.3)	.759
Type 2 diabetes mellitus	29 (33.0)	6 (27.3)	
Treatment with insulin	14 (17.1)	3 (14.3)	
Decreased bone density, n (%)			.714
Osteopenia	34 (44.2)	8 (42.1)	
Osteoporosis	13 (16.9)	2 (10.5)	
Fragility fracture within the past 12 months, n (%)	12 (13.6)	5 (23.8)	.250
Hyperlipidemia, n(%)	69 (78.4)	18 (90.0)	.237
Atherosclerotic cardiovascular disease, n(%)	7 (8.0)	5 (22.7)	.047
Venous thromboembolic event in the last 12 months, n (%)	5 (5.7)	1 (4.5)	.834
Weight gain, n (%)	74 (84.1)	15 (68.2)	.089
Truncal obesity, n (%)	66 (75.0)	17 (77.3)	.825
Supraclavicular and/or dorsocervical fat accumulation, n (%)	62 (70.5)	16 (72.7)	.834
Rounding of face ± plethora, n (%)	63 (71.6)	16 (72.7)	.916
Skin changes (violaceous striae, thinning, and/or bruising), n (%)	66 (75.0)	20 (90.9)	.106
Proximal muscle weakness (self-reported), n (%)	59 (67.0)	17 (77.3)	.353
Clinical severity score	15.0	15.5	.515
Median (Q1-Q3)	(9.0-17.0)	(12.2-16.8)	
Clinical severity score, n (%)			.446
Low	18 (20.5)	2 (9.1)	
Medium	34 (38.6)	9(40.9)	
High	36 (40.9)	11 (50.0)	
**Biochemical and radiological characteristics**			
ACTH, pg/mL, available for n = 109	17.0	14	.679
Median (Q1-Q3)	(5.7-65.0)	(9.3-53.5)	
DHEA-S, mcg/dL, available for n = 101	62.0	80.5	.605
Median (Q1-Q3)	(34.0-131.0)	(44.8-166.2)	
1-mg DST, mcg/dL, available for n = 92	6.8	3.5	.268
Median (Q1-Q3)	(3.0-15.1)	(2.5-11.9)	
8-mg DST, mcg/dL, available for n = 31	2.8	3.5	.600
Median (Q1-Q3)	(1.8-6.1)	(1.6-5.2)	
24-hour urine cortisol, μg/24 hours, available for n = 83	93.0	86.5	.558
Median (Q1-Q3)	(43.0-265.1)	(66.3-212.2)	
Late-night salivary cortisol, ng/dL, available for n = 63	225.5	184	.931
Median (Q1-Q3)	(129.2-405.8)	(92.0-427.0)	
Biochemical severity score	7.0	6	.674
Median (Q1-Q3)	(4.0-10.0)	(4.0-7.8)	
Biochemical severity score, n (%)			.153
Low	27 (30.7)	7 (31.8)	
Medium	20 (22.7)	9 (40.9)	
High	41 (46.6)	6 (27.3)	
**Glucocorticoid dose postsurgery**			
Initial daily hydrocortisone equivalent dose, mg, available for n = 96	50.0	40.0	.238
Median (Q1-Q3)	(40.0-50.0)	(40.0-50.0)	
Week 12 daily hydrocortisone equivalent dose, mg, available for n = 82	20.0	20.0	.091
Median (Q1-Q3)	(20.0-20.0)	(20.0-20.0)	
Glucocorticoid type used during 12 weeks postsurgery, n (%)			
Hydrocortisone alone	37.0 (42.0)	8.0 (36.4)	.785
Prednisone alone	29.0 (33.0)	9.0 (40.9)	
Hydrocortisone and prednisone	13.0 (14.8)	2.0 (9.1)	
No glucocorticoid	9.0 (10.2)	3.0 (13.6)	
Recovery of adrenal function during 12 weeks postsurgery, n (%), available for n = 96	13.0 (14.8)	2.0 (9.1)	.771

The Wilcoxon rank sum test was used to compare continuous measurements and the chi-square test was used to compare categorical measurements.

Abbreviations: ACTH, adrenocorticotropic hormone; BMI, body mass index; DHEA-S, dehydroepiandrosterone sulfate; DST, dexamethasone suppression test; MACS, mild autonomous cortisol secretion.

**Table 2.** Quality of life before and 12 weeks after surgical remission of hypercortisolism in patients using MUSE vs referents

Variables	Baseline			12 weeks postsurgery		
	Referent cohort	MUSE cohort	*P* value*^a^*	Referent cohort	MUSE cohort	*P* value*^a^*
n	82	22		82	22	
**SF-36, Z score, mean (SD)**						
Physical Component Summary score	35.9 (1.45)	33.8 (2.80)	.502	34.0 (0.92)	38.1 (1.77)	.**041**
Mental Component Summary score	39.2 (1.29)	32.8 (2.48)	.**022**	42.1 (1.16)	40.8 (2.26)	.624
**CushingQoL, Z score, mean (SD)**						
Physical score	33.5 (2.63)	25.0 (5.04)	.136	51.9 (2.08)	49.0 (3.96)	.517
Psychosocial score	37.8 (2.42)	31.4 (4.61)	.226	46.0 (1.68)	45.0 (3.16)	.784
Overall Cushing score	36.9 (2.29)	29.8 (4.34)	.153	47.5 (1.58)	46.3 (2.96)	.726
**AddiQoL**						
AddiQoL total score	72.7 (1.50)	65.5 (2.90)	**0**.**031**	75.7 (1.08)	75.9 (2.11)	.941

At 12 weeks after successful surgery for hypercortisolism, estimated marginal means are shown from analysis of covariance models adjusting for baseline values. Values are expressed as means (standard error). SF-36 and CushingQoL scores were standardized to range from 0-100, with higher scores corresponding with higher self-reported quality of life.

Abbreviations: QOL, quality of life; SF-36, 36-item Short Form Health Survey.

^a^Two-sample *t* test was used to compare measurements at presurgical baseline.

Among patients offered MUSE, there were no significant differences in the baseline characteristics or quality of life between patients compliant with MUSE for > 6 weeks and those who used it for < 6 weeks (Table 3 and 4).

**Table 3.** Baseline characteristics for the MUSE participants based on compliance

Characteristic	Compliant with MUSE	Noncompliant with MUSE	*P* value
n	22	30	
**Demographic characteristics**			
Age at baseline, years	53.2	49.2	.133
Median (Q1-Q3)	(44.5-63.2)	(37.4-55.2)	
Women, n (%)	17 (77.3)	27 (90.0)	.209
White race, n (%)	20 (90.9)	27 (90.0)	.913
**Clinical assessment and physical examination finding**			
BMI, kg/m^2^	31.4	33.4	.679
Median (Q1-Q3)	(26.1-40.1)	(26.9-37.2)	
Type of hypercortisolism, n (%)			.598
MACS	8 (36.4)	6 (20.0)	
Adrenal Cushing	4 (18.2)	7 (23.3)	
Pituitary Cushing	9 (40.9)	16 (53.3)	
Ectopic Cushing	1 (4.5)	1 (3.3)	
Duration of symptoms before diagnosis, months	15.0	12.0	.584
Median (Q1-Q3)	(4.2-69.0)	(12.0-48.0)	
Hypertension, n (%)	19 (86.4)	21 (70.0)	.166
Hypertension treated with 3 or more antihypertensives, n (%)	7 (31.8)	9 (30.0)	.889
Hyperglycemia, n (%)			.667
Prediabetes	6 (27.3)	11 (37.9%)	.959
Type 2 diabetes mellitus	6 (27.3)	8 (27.6%)	
Treatment with insulin	3 (14.3)	4 (14.8%)	
Decreased bone density, n (%)			.630
Osteopenia	8 (42.1)	13 (52.0)	
Osteoporosis	2 (10.5)	1 (4.0)	
Fragility fracture within the past 12 months, n (%)	5 (23.8)	2 (6.7)	.083
Hyperlipidemia, n(%)	18 (90.0)	22 (73.3)	.149
Atherosclerotic cardiovascular disease, n(%)	5 (22.7)	4 (13.3)	.376
Venous thromboembolic event in the last 12 months, n (%)	1 (4.5)	0 (0.0)	.238
Weight gain, n (%)	15 (68.2)	26 (86.7)	.107
Truncal obesity, n (%)	17 (77.3)	28 (93.3)	.094
Supraclavicular and/or dorsocervical fat accumulation, n (%)	16 (72.7)	21 (70.0)	.830
Rounding of face ± plethora, n (%)	16 (72.7)	25 (83.3)	.355
Skin changes (violaceous striae, thinning, and/or bruising), n (%)	20 (90.9)	23 (76.7)	.180
Proximal muscle weakness (self-reported), n (%)	17 (77.3)	20 (66.7)	.404
Clinical severity score	15.5	13.5	.618
Median (Q1-Q3)	(12.2-16.8)	(11.0-17.0)	
Clinical severity score, n (%)			.655
Low	2 (9.1)	5 (16.7)	
Medium	9 (40.9)	13 (43.3)	
High	11 (50.0)	12 (40.0)	
**Biochemical and radiological characteristics**			
ACTH, pg/mL	14.0	37.5	.577
Median (Q1-Q3)	(9.3-53.5)	(11.0-58.0)	
DHEA-S, mcg/dL, available for n = 48	80.5	94.5	.610
Median (Q1-Q3)	(44.8-166.2)	(39.5-152.2)	
1 mg DST, mcg/dL, available for n = 43	3.5	6.8	.483
Median (Q1-Q3)	(2.5-11.9)	(3.0-9.9)	
8 mg DST, mcg/dL, available for n = 16	3.5	2.8	.211
Median (Q1-Q3)	(1.6-5.2)	(1.4-3.3)	
24-hour urine cortisol, μg/24 hours, available for n = 39	86.5	79.0	.573
Median (Q1-Q3)	(66.3-212.2)	(47.0-153.0)	
Late-night salivary cortisol, ng/dL, available for n = 36	184.0	147.0	.895
Median (Q1-Q3)	(92.0-427.0)	(88.5-259.0)	
Biochemical severity score	6.0	6.0	.777
Median (Q1-Q3)	(4.0-7.8)	(3.2-9.0)	
Biochemical severity score, n (%)			.593
Low	7 (31.8)	9 (30.0)	
Medium	9 (40.9)	9 (30.0)	
High	6 (27.3)	12 (40.0)	
**Glucocorticoid dose postsurgery**			
Initial daily hydrocortisone equivalent dose, mg, available for n = 42	40.0	40.0	.243
Median (Q1-Q3)	(40.0-50.0)	(36.8, 50.0)	
Week 12 daily hydrocortisone equivalent dose, mg, available for n = 36	20.0	20.0	.310
Median (Q1-Q3)	(20.0-20.0)	(20.0-20.0)	
Glucocorticoid type used during 12 weeks postsurgery, n (%)			.829
Hydrocortisone alone	8 (36.4)	13 (46.4)	
Prednisone alone	9 (40.9)	8 (28.6)	
Hydrocortisone and prednisone	2 (9.1)	3 (10.7)	
No glucocorticoid	3 (13.6)	4 (14.3)	
Recovery of adrenal function during 12 weeks postsurgery, n (%)	2 (9.1)	5 (17.9)	.660

The Wilcoxon rank sum test was used to compare continuous measurements and the chi-square test was used to compare categorical measurements.

Abbreviations: ACTH, adrenocorticotropic hormone; BMI, body mass index, DHEA-S, dehydroepiandrosterone sulfate; DST, dexamethasone suppression test; MACS, mild autonomous cortisol secretion.

**Table 4.** Quality of life in patients offered MUSE intervention

Variables	Baseline		
	Compliant with MUSE	Noncompliant with MUSE	*P* value*^a^*
n	22	29	
**SF-36, Z score, mean (SD)**			
Physical Component Summary Score	33.8 (13.1)	32.6 (12.3)	.739
Mental Component Summary Score	32.8 (10.4)	35.3 (10.8)	.403
**CushingQoL, Z score, mean (SD)**			
Physical Score	25.0 (20.4)	27.1 (19.1)	.712
Psychosocial Score	31.4 (15.5)	28.9 (18.5)	.611
Overall Cushing Score	29.8 (15.2)	28.2 (16.3)	.716
**AddiQoL**			
AddiQoL total score	65.5 (12.4)	67.6 (11.6)	.555

Values are expressed as means (SD). SF-36 and CushingQoL scores were standardized to range from 0-100, with higher scores corresponding with higher self-reported quality of life.

Abbreviations: QOL, quality of life; SF-36, 36-item Short Form Health Survey.

^a^Two-sample *t* test was used to compare measurements at presurgical baseline. Values are expressed as means (SD). SF-36 and CushingQoL scores were standardized to range from 0-100, with higher scores corresponding with higher self-reported quality of life.

### Postsurgery

In the 22 patients compliant with MUSE for > 6 weeks, participants practiced guided meditation using MUSE for a median of 49 days (Q1-Q3: 27-67) and utilized MUSE at least once per week for a median of 11 weeks (9-12), Table 5. MUSE was used at least 3 separate days per week for a median of 8 weeks (Q1-Q3: 5-10), Table 5.

**Table 5.** Utilization of MUSE by the MUSE cohort participants during week 1 to week 12 after surgery

N of participants	22
Overall duration of MUSE utilization, hours Median (Q1-Q3)	9.3 (4.5-15.5)
Days when MUSE was used, n Median (Q1-Q3)	49 (27-66)
Weeks when MUSE was used at least once, n Median (Q1-Q3)	11 (9-12)
Weeks when MUSE was used on at least 3 separate days, n Median (Q1-Q3)	8 (5-10)

**Figure 2.**
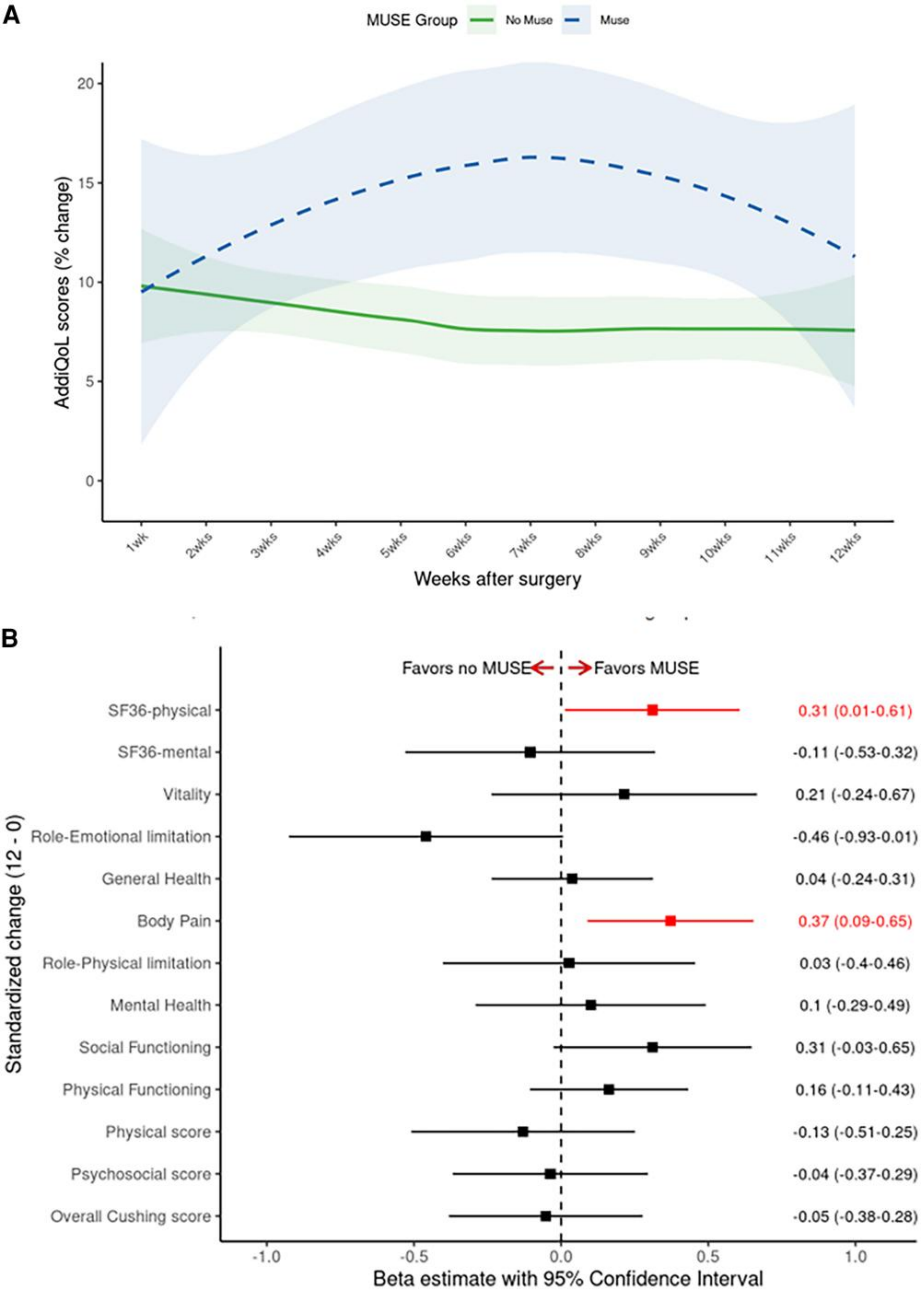
Panel A, Trajectory of withdrawal symptoms as measured by AddiQoL score change within 12 weeks after surgery in the MUSE vs referent cohort. Note: In comparison to the referent group, MUSE group had similar increase in the AddiQoL score during weeks 5-8 postsurgery (beta of 7.7 (95% CI of −0.3 to 16), *P* = .06) and during week 9-12 postsurgery (beta of 5.3 (95% CI of −2.6 to 13), *P* = .2). Panel B, Standardized change in the quality-of-life assessments between 12 weeks and baseline assessments. Note: All assessments were adjusted for baseline quality-of-life assessments. SF-36 physical component quality of life and SF-36 Body Pain subdomain demonstrated a higher improvement in patients using MUSE vs patients not using MUSE. No other differences were noted between MUSE and non-MUSE groups.

Although the MUSE cohort at baseline had a lower AddiQoL score and SF-36 mental component summary score (reflective of worse quality of life), the differences in SF-36 mental component score (mean 37.8, SD 9.3 vs 42.9, SD 13.2; *P* = .090) and AddiQoL score (mean 72.0, SD 10.3 vs 76.8, 14.3; *P* = .142) were no longer present between the groups at the 12-week follow-up (Table 2). The trajectory of the AddiQoL score over 12 weeks after surgery was no different between the 2 groups (Fig. 2A). The MUSE cohort demonstrated higher (improved) SF-36 physical component score at the 12-week follow-up (Table 2, Fig. 2B).

The multivariable analysis of age, sex, BMI, clinical severity score, baseline quality of life assessment, and the use of MUSE demonstrated that the use of MUSE was an independent predictor of a better SF-36-physical score at 12 weeks postsurgery (beta 4.2, 95% CI: 0.5-7.9, *P* = .026), **Table 6**.

**Table 6.** Multivariable analysis of quality-of-life measures at 12 weeks postsurgery for hypercortisolism

Variables	Assessments at 12 weeks postsurgery									
	AddiQoL		SF-36 PCS		SF-36 MCS		CushingQoL Physical		CushingQoL Psychosocial	
	Beta (95% CI)	*P* value	Beta (95% CI)	*P* value	Beta (95% CI)	*P* value	Beta (95% CI)	*P* value	Beta (95% CI)	*P* value
Age (per 1 year increase)	0.02 (−0.13-0.16)	.8	0.20 (0.07-0.33)	.**004**	0.04 (−0.12-0.21)	.6	−0.08 (−0.37-0.20)	.6	0.17 (−0.06-0.40)	.2
Women (vs men)	−2.2 (−7.1-2.6)	.4	−3.5 (−7.4-0.41)	.078	0.99 (−4.3-6.3)	.7	−5.1 (−14.0-4.1)	.3	−4.6 (−12-3.0)	.2
BMI (per 1 kg/m^2^ increase)	0.11 (−0.14-0.36)	.4	0.04 (−0.18-0.25)	.7	0.15 (−0.14-0.43)	.3	−0.60 (−1.1-0.06)	.**029**	0.36 (−0.05-0.76)	.088
MUSE (vs no MUSE)	0.41 (−4.2-5.0)	.9	4.2 (0.50-7.9)	.**026**	−1.3 (−6.4-3.9)	.6	−2.8 (−12-6.0)	.5	−0.80 (−7.7-6.1)	.8
Clinical severity score (per 1 unit increase)	−0.52 (−0.97- -0.06)	.**025**	−0.25 (−0.64-0.14)	.2	0.03 (−0.48-0.54)	>.9	0.60 (−0.36-1.6)	.2	−0.75 (−1.5-0.03)	.**041**
Assessment at baseline (presurgery)	0.63 (0.48- 0.78)	**<**.**001**	0.50 (0.35-0.65)	**<**.**001**	0.61 (0.42-0.79)	**<**.**001**	0.55 (0.37-0.74)	**<**.**001**	0.53 (0.36-0.69)	**<**.**001**

Abbreviations: QOL, quality of life; SF-36, 36-item Short Form Health Survey.

## Discussion

In this pilot study investigating meditation use during recovery from hypercortisolism, we show that MUSE was associated with improvement in the physical component of SF-36. We found that when offered, 42% of participants used MUSE for at least 6 weeks within 12 weeks postsurgery. We found no differences in the demographic, clinical, or quality of life characteristics in patients compliant with MUSE vs those who used MUSE < 6 weeks.

MUSE is a portable, wearable, wireless, headband-style EEG device for guided meditation and has been reported to improve quality of life, stress, and fatigue in women with recently diagnosed breast cancer within 3 months after surgery. In this pilot study of MUSE intervention in patients recovering from endogenous hypercortisolism, we also show that MUSE was associated with a higher degree of improvement in the physical component of SF-36, after adjusting for important factors: age, sex, BMI, clinical severity, and baseline quality of life. We have not found that MUSE had an impact on other quality-of-life assessments, including the mental component of SF-36, CushingQoL physical or psychosocial score, or AddiQoL score. Possible explanations include a small sample size, especially considering the heterogeneity of presentation of patients with endogenous hypercortisolism and lower than recommended frequency and duration of MUSE use (even in those who met criteria for compliance).

Only 42% of patients offered MUSE used it for more than 6 of 12 weeks after surgery. As we have not found differences between the baseline demographics, clinical presentation, or quality of life between compliant vs noncompliant patients, some of the potential explanations for lower use include technical issues with the phone-MUSE connection, uncomfortable fit of the EEG headband, and work schedule/other responsibilities interfering with use.

Strengths of this study include prospective enrollment with longitudinal follow-up, use of standardized glucocorticoid protocols, and use of validated surveys. The MUSE and referent groups were matched for possible confounding factors such as demographics, hypercortisolism type, BMI, and postsurgical glucocorticoid therapy. The 2 groups had similar baseline prevalence and severity of comorbidities and similar clinical and biochemical clinical severity scores. To understand the reason influencing MUSE use among patients, we compared the baseline characteristics and quality of life between patients using MUSE for < 6 weeks and > 6 weeks and found no significant differences. Multivariable analysis was predefined to account for important factors influencing quality of life. Limitations of our study include small sample size of the MUSE cohort, referral bias, predominance of White study participants which limits generalizability of the results to more diverse populations, and reliance on participant ability to address at home technical issues with the MUSE device. Notably, despite the matching and nonselective approach to MUSE enrollment, patients in the MUSE cohort had lower baseline quality of life. We addressed this limitation by adjusting our analyses for the baseline quality-of-life assessment. In this pilot study, we were not able to evaluate the differential impact of MUSE based on hypercortisolism subtype due to small sample size. Another limitation is lack of additional engagement with the MUSE participants, which likely decreased compliance with MUSE use. However, a more intense interaction with study participants may have impacted patient's quality of life and confounded our results.

In conclusion, in this pilot study of patients with endogenous hypercortisolism treated with surgery, we showed that meditation intervention is associated with a higher degree of improvement in the postsurgical physical component of SF-36 quality-of-life assessment. Counseling patients with endogenous hypercortisolism about the use of meditation after surgery may improve symptoms of GWS and accelerate recovery.

## Data Availability

Some or all datasets generated during and/or analyzed during the current study are not publicly available but are available from the corresponding author on reasonable request.

## Abbreviations

BMI: body mass index
EEG: electroencephalography
GWS: glucocorticoid withdrawal syndrome
MACS: mild autonomous cortisol secretion
SF-36: 36-item Short Form Health Survey
